# Evidence for simple volcanic rifting not complex subduction initiation in the Laxmi Basin

**DOI:** 10.1038/s41467-020-16569-y

**Published:** 2020-06-01

**Authors:** Peter D. Clift, Gérôme Calvès, Tara N. Jonell

**Affiliations:** 1 Department of Geology and Geophysics, E235 Howe-Russell, Louisiana State University, Baton Rouge, LA 70803 USA; 2 Université Toulouse III, GET-OMP, 14 Avenue Edouard Belin, 31400 Toulouse, France; 3 School of Earth and Environmental Sciences, The University of Queensland, St. Lucia, QLD 4072 Australia

**Keywords:** Geochemistry, Geophysics, Tectonics

## Abstract

Recently, Pandey et al proposed relict subduction initiation occurred along a passive margin in the northwest Indian Ocean. Here, Clift et al question the evidence for subduction initiation, suggesting that simpler rifting-related processes can more simply explain the available data for the Laxmi Basin.

**Arising from** Pandey et al. *Nature Communications* 10.1038/s41467-019-10227-8 (2019)

In their recent paper Pandey et al.^1^ use geochemical data from an International Ocean Discovery Program (IODP) site in the Laxmi Basin to propose a previously unrecognized proto-subduction zone in the eastern Arabian Sea in the region of Laxmi Basin (LB), offshore western India, during the Late Cretaceous-Early Cenozoic. A short section of basaltic basement (~16 m penetration) was recovered at Site U1457 on the eastern flank of Laxmi Ridge (LR) (Fig. 1). The geochemistry of the volcanic rocks exhibited subduction-related characters, such as relative Nb and Ta depletions, while Nd and Sr isotopes indicate some involvement of continental material during petrogenesis, interpreted as the incorporation of subducted sediment within mantle melts. On the basis of these data this study proposes a significant revision of the Cretaceous-Cenozoic plate motions involving complex shearing and rotation of blocks during the opening of the Indian Ocean. However, we here argue that the primary lines of evidence used in this study are misinterpreted and that other data sets in the region, not accounted for by these authors, render this new model untenable.

**Fig. 1:**
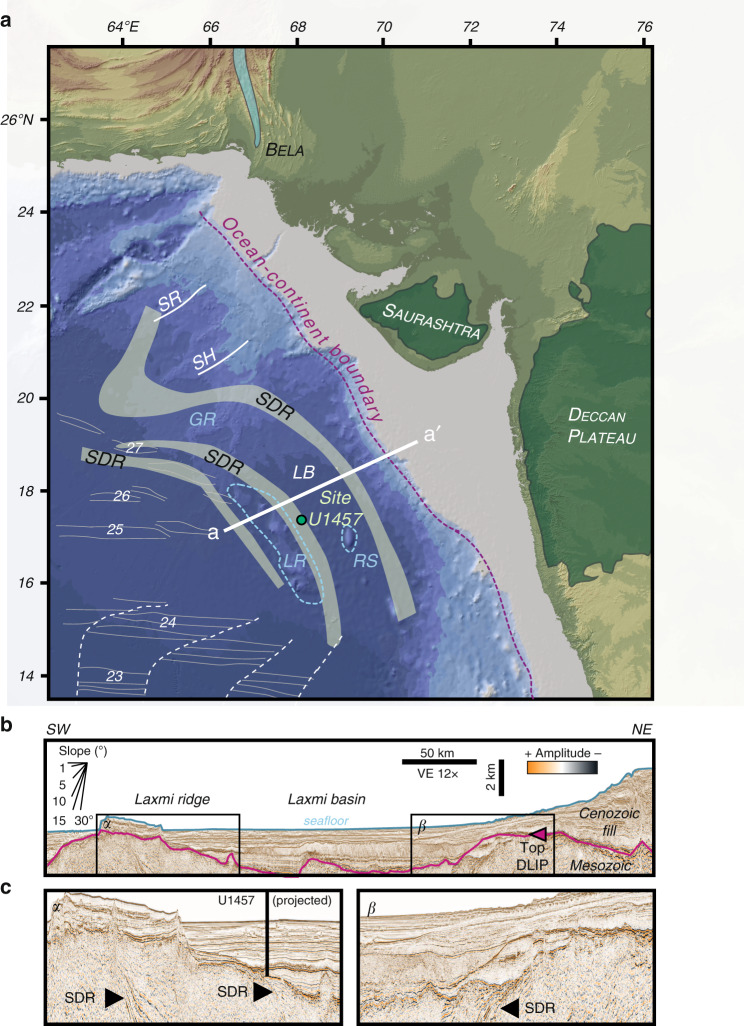
Location and Geophysical Images. **a** Shaded bathymetric map of the Arabian Sea and surrounding regions, showing the location of IODP Site U1457, proposed continent-ocean boundaries (yellow dashed lines), the extent of known seaward dipping reflectors from Calvès et al^4^. Magnetic anomalies (thin grey numbered lines) and transform fault (dashed white lines). SDR seaward-dipping reflector, SR Somnath Ridge, SH Saurashtra High, GR Gop Rift, LR Laxmi Ridge, LB Laxmi Basin, RS Raman Seamount. **b** Seismic reflection profile across LR and LB showing presence of SDRs, position of ocean-continent boundary (OCB) and the Deccan Large Igneous Province (DLIP). c) Close-up of LR showing east-dipping SDRs in α and β.

Exactly where a LB proto-trench would have been located is not clear, nor how the LR, presumably representing a proto-forearc massif, was involved. In contrast, most studies consider the LR to be a rifted continental fragment derived from the Indian margin. In the model proposed by Pandey et al.^1^ the LR is required to be oceanic crust. Pandey et al.^1^ improperly cite the study by Krishna et al.^2^ who interpret the LR to be stretched and thinned continental lithosphere. A continental origin for the LR, albeit with magmatic underplating related to excess melting above a plume prior to opening of the LB, was also inferred from seismic refraction data^3^. The new model^1^ further ignores the fact that the LR is part of a volcanic margin sequence running south from two well-defined volcanic ridges, the Somnath Ridge and the Saurashtra High, which together form a pre‐Deccan continental flood basalt province (∼75–65.5 Myr ago)^4^. Indeed, Calvès et al.^4^ report the presence of seaward-dipping reflectors on either side of LB that represent subaerial volcanic flows erupted during the initial extension of the basin (Fig. 1b, c). The presence of seaward-dipping reflectors is incompatible with the presence of a proto-subduction zone but is a classic signature of the more widely accepted volcanic rifted margin setting^5^. The shallower than normal oceanic crust depths to basement in Laxmi Basin (2.5–2.7 km sediment unloaded modern depths in the region of the drilling sites^6^ compared with 5.2 km for 66 Myr old normal crust^7^) does not imply presence of subduction-related crust but simply continental crust that has not been extended to the degree required for seafloor spreading to occur (i.e., the crust is thicker than normal oceanic crust and composed of less dense material). Indeed, a rifted origin was proposed by the first two authors of the Pandey et al.^1^ study based on simple flexural extension models^6^ and despite the fact that it is incompatible with the subduction model they concurrently advocate in this second study. This more conventional approach to the origin of LB is moreover predicted by restoration of the flat-topped Raman Seamount at 66 Myr ago to close to sealevel. This prediction is consistent with the seamount morphology, implying a wave-cut top. A typical thermal subsidence model does a good job of matching the vertical tectonic constraints. Similarly, subsidence analysis of the LR shows a thermal subsidence history compatible with regular oceanic crust, albeit one affected by dynamic support from the Réunion plume in the early stages of basin rifting^8^. If this region is really an extinct forearc massif then this match would be purely coincidental.

The most important line of evidence supporting the Pandey et al.^1^ model is the subduction signature of the volcanic basement. Although lavas erupted in the earliest stages of subduction show very little subduction imprint^9^, the same is true of rocks from mid-ocean ridge or rifted margin settings. Many Tethyan passive margin volcanic rocks contain hints of a subduction-linked petrogenesis but are not generated above a subduction zone^10^. The same can be said of the late Oligocene to Recent rift volcanic rocks of the western United States^11^ where subduction signatures are proposed to reflect earlier, pre-rift enrichment and modification of the lithospheric mantle. These melts are then remobilized during rifting.

We use multi-element spider diagrams (Fig. 2) normalized to normal mid ocean basalt (N-MORB) values to highlight the fact that water-mobile elements, such as U, Pb and Ba are strongly enriched compared to water-immobile elements, although Sr is slightly depleted and K and Rb are close to N-MORB values. Enrichment in water mobile elements is a feature of subduction magmatism but may also indicate alteration. Lack of enrichment in some water-mobile elements is not typical of subduction-related magmatism. If Sr concentrations are affected by alteration then Sr isotopic values cannot be considered original and magmatic. Alteration would make the Laxmi volcanic rocks shift towards an appearance of subduction influence in the Pandey et al.’s ε_Nd_ vs ^87^Sr/^86^Sr isotope plot and away from the original MORB-like composition. More detailed discussion of the isotopes was not possible because the data were not made available on request. Furthermore, subduction magmatism is usually associated with relative depletions of Nb and Ta relative to other water-immobile elements, such as La and Ce, yet these depletions are not observed (Fig. 2a). Indeed, Ta is relatively enriched. We note that the positive relative Zr and Hf enrichments in the Laxmi Basin volcanic rocks are commonly recorded in Mariana forearc boninites^9^, but are not recorded in forearc basalts of the type favored by Pandey et al.

**Fig. 2:**
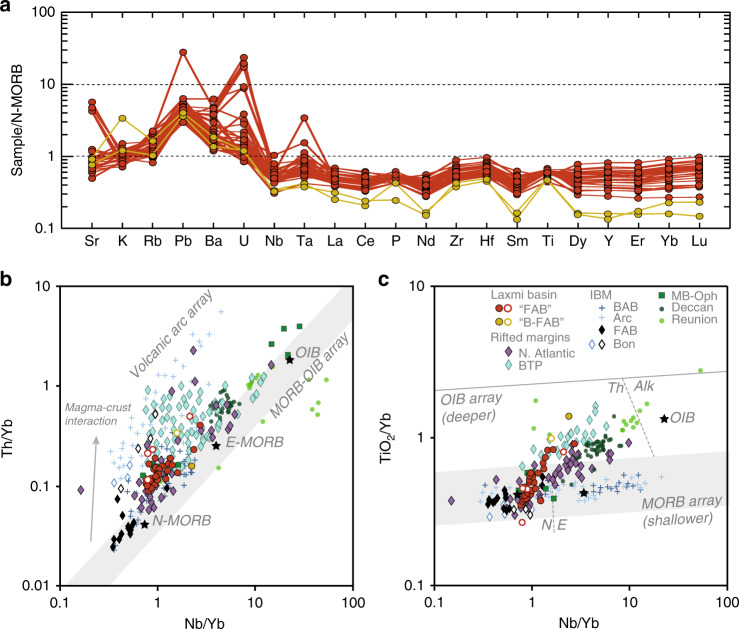
Trace element chemistry. **a** N-MORB normalized multi-element spider diagrams for the LB lavas. Water-mobile elements are plotted left of Ba, with immobile elements on the right side of the plot starting at Nb. Compatibility in mantle phases increases to the right within the immobile elements. N-MORB values are from Gale et al.^15^. **b** Nb/Yb–Th/Yb discrimination diagram^16^ highlights variable input from subduction, AFC processes, inheritance, and mantle source enrichment that can displace rock compositions from and along the MORB-OIB array. Data were filtered prior to plotting to remove fractionated samples with >55 wt% SiO_2_, significant crystallized oxides (Ti/Ti* < 0.85) and strong crustal contamination (Th/Nb > 0.2) following Pearce et al.^16^. Open symbols identify LB lavas and IBM boninites that do not meet these requirements. Analytical uncertainties are within the size of the plotted symbols. Negative Nb anomalies, common in rocks formed in subduction zones, result in compositions to be displaced sub-vertically from MORB. Many basalts unrelated to subduction can carry apparent subduction signatures as a result of crustal assimilation and inheritance and may similarly plot in the volcanic arc array. **c** The Nb/Yb–TiO_2_/Yb diagram^16^ can further help discriminate intra-plate from plate margin basalts as it is less sensitive to crustal input. Volcanic rifted margins, LB lavas and the Réunion plume plot along a steep diagonal from the OIB to MORB array. Increased extension and attenuation of continental lithosphere drives compositions from the OIB to MORB field as depth of melting shallows. Subduction-influenced rocks, such as basalts and boninites from the IBM, that involve higher degrees of fluid-flux melting under MORB-like temperatures, remain and evolve along the MORB array. Field labels from Pearce et al.^16^. Data compiled from GEOROC, IBM^9^, and Nauret et al.^17^. Alk alkaline; BABB back-arc basin, B-FAB boninitic forearc basalt, Bon boninite, BTP British Tertiary Province, E enriched type, E-MORB Enriched Mid-Ocean Ridge Basalt, FAB forearc, IBM Izu-Bonin-Mariana, LB Laxmi basin, MORB mid-ocean ridge basalt, N normal type, N. Atlantic North Atlantic margin, OIB Ocean Island Basalt. Data plotted in these figures are provided in Supplementary Data 1 and from the Mendeley databank at 10.17632/723c5d795r.1.

The most relevant comparison with the LR are the rifted volcanic margins of the North Atlantic. Ocean Drilling Program (ODP) Site 917 offshore SE Greenland penetrated *a* > 900-m-thick sequence of volcanic rocks erupted during initial break-up, prior to the onset of seafloor spreading. The lower and middle lava sequences at Site 917 have clear subduction signatures that reflect remobilization of older melts, rather than nascent subduction initiating in the North Atlantic at that time^12^ (Fig. 2b). Likewise, volcanic rocks of the British Tertiary Province on the conjugate margin also show a subduction-type trend on discrimination diagrams (Fig. 2b, c). In this context the continental signature inferred from Sr isotope geochemistry of the LB samples would not represent sediment subduction but simply contamination of mantle melts rising through modified, extended continental lithosphere prior to eruption.

To generate melt within a subduction zone requires significant downflexing of hydrated material into the mantle. A conceptual model following that of Stern and Bloomer^13^ is indeed shown, yet there is no geophysical or bathymetric evidence to support a proto-subduction feature within the LB. Furthermore, the nature of subduction initiation along an oceanic transform^14^ elicits questions on the kinematics required to permit subduction in the LB. Considering the limited extension within the basin that correspondingly produced similarly aged and buoyant oceanic material across the proposed transform boundary, it is questionable if enough gravitational instability would have existed across any transform in this region to initiate spontaneous SW polarity subduction, depicted by Pandey et al.^1^. This lack of buoyancy contrast, lack of structural evidence supporting significant NE-SW compression that could otherwise induce subduction, and no supporting morphological evidence make an incipient subduction model along the western Indian passive margin physically improbable. In any case, seismic reflection profiles across the LR moreover show no indication of a transform margin along the western side of the ridge, as predicted^4^.

Instead, most evidence supports the Laxmi Basin forming an extensional basin with seaward-dipping reflectors and a normal thermal subsidence history^6^. The age of the basin, which has still not been demonstrated from the drilled volcanic rocks, places it firmly within a wider pre-Deccan volcanic province^4^. Some contamination by older subduction-related rocks is a common feature in magmas from rifted continental margins and does not require nascent subduction at the time of eruption, even assuming that the supposed subduction signature is not simply the product of post-eruption alteration. All geophysical and geochemical evidence put forth by Pandey et al.^1^ to argue incipient subduction along the western Indian margin can be more simply explained by a series of rifts following pure shear strain accommodation, accompanied by varying degrees of involvement from plume-related asthenosphere. Occam’s razor does not favour a complex subduction model of the type advocated when a simple volcanic rifting model also matches the data. The break-up of Gondwana and the opening of the Indian Ocean is unlikely to be as complex as suggested.

## Supplementary information

Description of Additional Supplementary Files

Supplementary Data 1

## Data Availability

The geochemical data plotted in Fig. 2 was compiled from literature and is available in Supplementary Data 1 and from the Mendeley databank at 10.17632/723c5d795r.1.
