# Supplementary material for: Evidence for simple volcanic rifting not complex subduction initiation in the Laxmi Basin
Source: Nat Commun. 2020 Jun 1;11:2733. doi: 10.1038/s41467-020-16569-y (PMC7264180; doi:10.1038/s41467-020-16569-y)
Supplement: Supplementary file 1 — Description of Additional Supplementary Files [file 41467_2020_16569_MOESM1_ESM.pdf]

Description of Additional Supplementary Files

File Name: Supplementary Data 1
Description: Compiled geochemistry used to make plots in Figure 2
